# Association between maternity waiting home stay and obstetric outcomes in Yetebon, Ethiopia: a mixed-methods observational cohort study

**DOI:** 10.1186/s12884-021-03913-3

**Published:** 2021-07-03

**Authors:** Anne K. Erickson, Safa Abdalla, Alice Serenska, Bete Demeke, Gary L. Darmstadt

**Affiliations:** 1 Stanford University School of Medicine, Stanford, CA USA; 2 Present address: Department of Obstetrics and Gynecology, University of Washington, Seattle, WA USA; 3 Department of Pediatrics, Stanford University School of Medicine, Stanford, CA USA; 4 Stanford University, Stanford, CA USA; 5 Project Mercy, Yetebon, Ethiopia

**Keywords:** Maternal health, Newborn health, Childbirth, Maternity waiting home, Three delays, Delivery complication

## Abstract

**Background:**

A strategy for reducing adverse pregnancy outcomes is the expanded implementation of maternity waiting homes (MWHs). We assessed factors influencing MWH use, as well as the association between MWH stay and obstetric outcomes in a hospital in rural Ethiopia.

**Methods:**

Data from medical records of the Glenn C. Olson Memorial Primary Hospital obstetric ward were cross matched with records from the affiliated MWH between 1 and 2011 to 31 March 2014. Poisson regression with robust variance was conducted to estimate the relative risk (RR) of childbirth complications associated with MWH use vs. non-use. Five key informant interviews of a convenience sample of three MWH staff and two users were conducted and a thematic analysis performed of social, cultural, and economic factors underlying MWH use.

**Results:**

During the study period, 489 women gave birth at the hospital, 93 of whom were MWH users. Common reasons for using the MWH were post-term status, previous caesarean section/myomectomy, malposition/malpresentation, and low-lying placenta, placenta previa, or antepartum hemorrhage, and hypertension or preeclampsia. MWH users were more likely than non-users to have had a previous caesarean Sec. (15.1 % vs. 5.3 %, *p* < 0.001) and to be post-term (21.5 % vs. 3.8 %, *p* < 0.001). MWH users were also more likely to undergo a caesarean Sec. (51.0 % vs. 35.4 %, *p* < 0.05) and less likely (*p* < 0.05) to have a spontaneous vaginal delivery (49.0 % vs. 63.6 %), obstructed labor (6.5 % vs. 14.4 %) or stillbirth (1.1 % vs. 8.6 %). MWH use (*N* = 93) was associated with a 77 % (adjusted RR = 0.23, 95 % Confidence Interval (CI) 0.12–0.46, *p* < 0.001) lower risk of childbirth complications, a 94 % (adjusted RR = 0.06, 95 % CI 0.01–0.43, *p* = 0.005) lower risk of fetal and newborn complications, and a 73 % (adjusted RR = 0.27, 95 % CI 0.13–0.56, *p* < 0.001) lower risk of maternal complications compared to MWH non-users (*N* = 396). Birth weight [median 3.5 kg (interquartile range 3.0-3.8) vs. 3.2 kg (2.8–3.5), *p* < 0.001] and 5-min Apgar scores (adjusted difference = 0.25, 95 % CI 0.06–0.44, *p* < 0.001) were also higher in offspring of MWH users. Opportunity costs due to missed work and need to arrange for care of children at home, long travel times, and lack of entertainment were suggested as key barriers to MWH utilization.

**Conclusions:**

This observational, non-randomized study suggests that MWH usage was associated with significantly improved childbirth outcomes. Increasing facility quality, expanding services, and providing educational opportunities should be considered to increase MWH use.

**Supplementary Information:**

The online version contains supplementary material available at 10.1186/s12884-021-03913-3.

## Background

Ethiopia’s burden of maternal and neonatal mortality is among the highest in the world. In response to this crisis, the Ethiopian government employed a number of initiatives to improve access to maternal and newborn care. Despite measures to eliminate associated fees and allocate resources toward health education and facility improvement, Ethiopian mothers and babies still suffer disproportionately high rates of obstetric complications and fatalities. An estimated 62 % of maternal deaths worldwide occurred in sub-Saharan Africa in 2017 [1], with 401 mothers dying per 100,000 live births in Ethiopia [2].

The three-delays model provides a framework for addressing the myriad difficulties expectant mothers in Ethiopia may face [3]. This framework highlights delays in making the decision to seek care, expeditious access to care, and receiving care. The most critical window for maternal and newborn medical intervention occurs closest to delivery, when pregnancy-related complications and logistical obstacles emerge. One approach to reducing potential delays in receiving skilled childbirth care, particularly emergency obstetric care, is the utilization of maternity waiting homes (MWHs) – residential structures situated near healthcare facilities where pregnant women can await labor. MWHs offer potential benefits to women at higher risk for pregnancy complications and those living in remote areas by providing streamlined access to care. Although skilled birth attendance has risen steadily since 2000, only 28 % of births in Ethiopia in 2016 had skilled birth attendance [4]. Where proximity to health centers and hospitals has been assured via MWHs, facility delivery rates among MWH users tend to be high – as high as 98.8 % in one study in Ethiopia [5]. Although MWH implementation can significantly increase access to facility childbirth care, they remain underutilized. In 2015, the Ethiopian Ministry of Health published guidelines for standardizing and expanding MWH use [6], yet the Ethiopian Public Health Institute found that MWH occupancy was dramatically lower than available capacity, averaging 29 % in 2016 [7].

Frequently cited factors determining MWH usage include the cost of travel [8–19] and lost wages [1, 8, 14, 18–22] during MWH stay, whether food is provided [15, 22–24], the need to care for other children [6, 24], and social support for the choice to use a MWH [1, 6, 14, 15, 20–22, 24, 25]. The balance of perceived need and benefits is a determining factor for women considering MWH use. Some research suggests that MWHs improved maternal and neonatal health outcomes [5, 26], while other studies have found that MWHs had no effect on those outcomes [16, 27]. These results are difficult to generalize, given the ongoing variation in MWH setup and the locally specific context in which each MWH operates [27, 28].

In this mixed-methods observational cohort study, we sought to describe women’s use of a MWH associated with a rural hospital in Ethiopia, including a quantitative assessment of childbirth outcomes and qualitative commentary from patients and providers on the factors impacting MWH use and acceptability.

## Methods

### Study context

This study analyzed data from the record books of the obstetric ward of the Glenn C. Olson Memorial Primary Hospital in rural Yetebon, Ethiopia, and its affiliated MWH. The 52-bed hospital is located in a community of about 70,000 population comprised mostly of Gurage ethnic minority, Muslim inhabitants at the base of Mount Gurage in southwest Ethiopia. The hospital is operated by Project Mercy, a non-profit organization which has continually run a holistic set of health, education, and development programs in the area since 1993. The MWH is located a short walking distance from the hospital and can accommodate up to eight women at a time, including provision of a bed, linens, water, meals and emergency transportation.

### Study data

Overlapping data were available for both the hospital’s obstetric ward and the MWH from 1 to 2011, when MWH records began to be systematically maintained, to 31 March 2014, when the study period ended at the time of AKE’s departure from Yetebon. All women who delivered in the hospital and/or used the MWH during this time period and had childbirth outcome data available in the obstetric logbook of the hospital were eligible for inclusion in the study. Data in the obstetric logbook included hospital identification number; maternal age; delivery date; delivery type (caesarean section, spontaneous vaginal delivery, vacuum or forceps-assisted delivery); the baby’s sex, weight, and Apgar scores; notes on complications and risk factors (fetal and maternal), and maternal human immunodeficiency virus (HIV) status. Data in the MWH logbook included hospital identification number, MWH arrival and departure date, delivery date, delivery type, baby’s sex, and notes by MWH staff in consultation with the hospital’s physician/surgeon on fetal and maternal complications and risk factors. These notes included the reason(s) for referral/presentation to the MWH (more than one reason was possible) and the woman’s birth outcome. Reasons for MWH use recorded in the logbook focused on biomedical, and not socio-economic reasons.

Women who were recorded in the obstetric logbook in the hospital – including childbirth outcome data – and who used the MWH comprised the “treatment” or exposure arm, which we also identified as “MWH users.” Women who were recorded in the obstetric logbook in the hospital – including childbirth outcome data – and did not use the MWH were considered comparison women, and were designated “MWH non-users”.

### Data management

Data from both logbooks were entered into Microsoft Excel and uploaded into Stata 14.0 (StatCorp LLC, College Station, Texas, USA) for analysis. Using each woman’s unique hospital identification number, women in MWH records were matched against the obstetric record. These matches were then individually checked to ensure accuracy. Three variables which were recorded in both logbooks – baby’s sex, delivery type, and delivery date – were used to verify record match. For MWH logbook records without an obstetric logbook match, every effort was made to identify the outcome of the MWH stay through further examination of the MWH logbook and the patients’ original paper medical charts, which were kept in storage on hospital premises.

Data were assessed for missing values. When possible, missing values were imputed from MWH records to obstetric records, or vice versa. Inconsistencies in the data were resolved in favor of the obstetric record, because this record was more standardized and long-standing.

Recorded complications or indications for MWH use were grouped into medically relevant categories. For example, a category termed “Obstructed or prolonged labor” was created to include records which noted the complications “cephalo-pelvic disproportion,” “obstruction,” or “cervical arrest.” A category termed “malposition or malpresentation” included complications such as “breech,” “hand presentation,” and “transverse lie.”

### Data analysis

We first generated descriptive statistics on the MWH users and non-users who gave birth at the hospital. We then conducted univariate statistical tests (Chi-squared, Fisher’s exact, and two-sample unpaired t-tests, as appropriate) to assess differences in the characteristics of MWH users and non-users and their risk factors recorded in the hospital’s obstetric logbook according to maternal age, multiple pregnancy, HIV status, preeclampsia, fetal risk factors (anomalies, malposition), placenta previa, post-term, and delivery type. Next, we synthesized childbirth complications into three outcome categories: any complication, any fetal or newborn complication, and any maternal complication. Any fetal or newborn complication was defined as the reporting of stillbirth/intrauterine fetal death, fetal distress, preterm birth, or death after delivery. Any maternal complication was defined as the recording of, antepartum hemorrhage, postpartum hemorrhage, premature rupture of membranes, obstruction of labor, or uterine rupture. Any complication was a summary variable for any of those reported complications. We disregarded complications recorded in both the MWH record and obstetric record because those would have been present before labor and thus were not considered childbirth complications (any complication, *N* = 4; any fetal or newborn complication, *N* = 3; any maternal complication, *N* = 1). We performed a sensitivity test of the impact of this assumption by repeating the analysis, keeping those complications. We used Poisson regression with robust variance to measure the relative risk (RR) of complications associated with MWH use or non-use. We also compared the 1-min and 5-min Apgar scores of live births between the two groups with a generalized additive model.

Figure 2 shows the conceptual models that informed decisions about adjustments for covariates. Due to insufficient documentation of the circumstances of delivery or the risk factor profile of women who did not attend the MWH, we treated the mode of delivery in two ways. In conceptual model A, birth by caesarean section was considered a mediator. In conceptual model B, caesarean section was considered a proxy for undocumented risk. Other covariates were multiple pregnancy, and maternal age. All covariates were tested for potential confounding or independent association with the outcome, and those showing independent associations that were significant at the 0.05 alpha cut-off level were kept in the model. Models were re-run without adjusting for caesarean section to implement conceptual model A and adjusting for caesarean section to implement model B. All models for Apgar score were adjusted for birth by caesarean section.

### Qualitative assessment

Since the MWH logbook did not record the social, cultural, economic, and logistical factors underlying MWH use, we conducted key informant interviews with a convenience sample of three hospital staff members involved in running the MWH (i.e., healthcare workers) as well as two women who used the MWH during the time of data collection. Depending on interviewee preference, interviews were audio-recorded and transcribed, or recorded in detailed note form by AKE. Interviews with the healthcare workers were conducted in English, as they were English-speaking. Interviews with the MWH users were conducted in Amharic and translated from Amharic to English by the bilingual healthcare workers. Interviews were open-ended and explored experiences working with the MWH, its role as part of the healthcare system, and thoughts about potential MWH improvements. Interview content was analyzed inductively for recurring themes using principles from grounded theory [29].

## Results

### Hospital and MWH study cohort

**Fig. 1:**
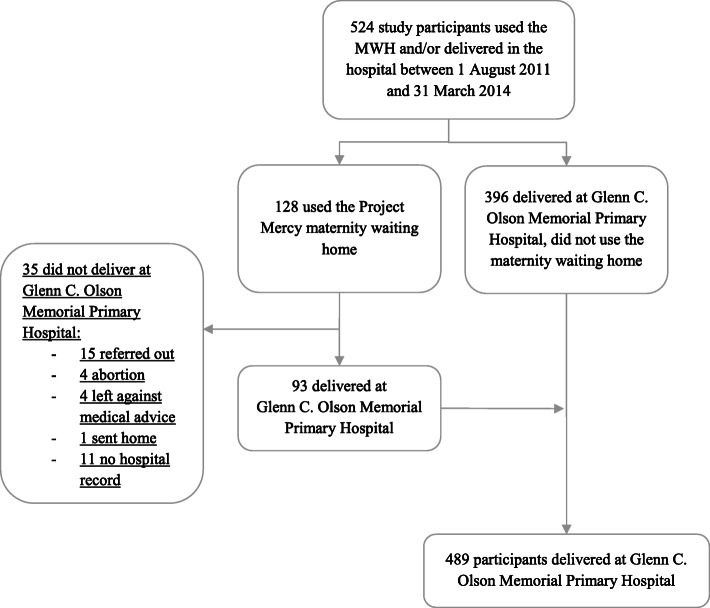
Study participant flow chart showing Project Mercy maternity waiting home and Glenn C. Olson Memorial Primary Hospital utilization in Yetebon, Ethiopia

A total of 524 women gave birth in the hospital and/or used the MWH during the study period, 489 of whom had childbirth outcome data available in the obstetric logbook of the hospital and thus were included in the study (Fig. 1). One hundred twenty-eight women used the MWH during the study period and were recorded in the MWH logbook. However, 35 of these 128 women did not give birth at the hospital or had missing hospital records and were excluded from the study, including 15 women who were referred to an outside hospital, four who attended the MWH for treatment of a missed or threatened abortion and not for childbirth, four who left the MWH against medical advice, and one who was sent home after monitoring. Eleven women were recorded as either having had a caesarean section (*N* = 8) or a spontaneous vaginal delivery (*N* = 3); however, we were not able to locate a corresponding obstetric record and these women were excluded. Thus, of the 489 women who presented to the hospital for childbirth and had birth outcome records during the study period, 93 used the MWH (72.6 % of 128 MWH users, 19.0 % of 489 births). The 489 women who gave birth at the hospital had 510 infants, 468 from singleton births and 42 from twin pregnancies. There were 463 live births and 47 stillbirths/intrauterine fetal deaths.

### Reasons for MWH use

Reasons for using the MWH included post-term status (*N* = 33), previous caesarean section or myomectomy (*N* = 24), malposition or malpresentation (*N* = 16), and low-lying placenta, placenta previa, or antepartum hemorrhage (*N* = 13) (Table 1). Although MWH staff noted that a woman’s remote location or barriers to hospital access were often reasons for MWH use, the MWH logbook listed only medical and not socio-cultural-economic reasons for MWH admission.

**Table 1:** Indications for use of the Project Mercy maternity waiting home (MWH) recorded in the MWH logbook, August 2011-March 2014 (*N* = 128), Yetebon, Ethiopia

Indication for MWH use	N (%)
Post-term	33 (25.8)
Previous caesarean section or myomectomy	24 (18.8)
Fetal malposition or malpresentation	16 (12.5)
Low-lying placenta, placenta previa, or antepartum hemorrhage	13 (10.2)
Hypertension or preeclampsia	6 (4.7)
Missed, incomplete, or threatened abortion	5 (3.9)
Other maternal problems (each < 5): grand multiparous mother, maternal medical problems (pyelonephritis, pneumonia, congestive heart failure), poor obstetric history (previous stillbirth), maternal pelvic deformity, maternal abdominal injury (admitted for observation)	11 (8.6)
Other fetal problems (each < 5): oligohydramnios, hydrocephalus, intrauterine growth restriction, fetal distress	6 (4.7)

### Characteristics and risk factors of women who gave birth at the hospital

We examined characteristics and risk factors of the cohort of 489 women who came to the hospital for childbirth, stratified by MWH use (Table 2). MWH users (*N* = 93) were slightly older than non-users (*N* = 396), with a median age of 29 years versus 27 years for MWH non-users (*p* < 0.05). Maternal HIV rates were low (*N* = 1 among MWH users, *N* = 6 in non-users; 1.4 % overall, with 98.4 % of results recorded), and were not significantly different between the two groups. Nearly three times the proportion of women giving birth via the MWH had previously undergone caesarean Sec. (14 of 93, 15.1 %) compared to non-users (21 of 396, 5.3 %) (*p* < 0.001), and in this setting, vaginal birth following a previous caesarean section was extremely rare. The proportion of women presenting post-term was approximately five-fold higher for MWH users (20 of 93, 21.5 %) compared to non-users (15 of 396, 3.8 %) (*p* < 0.001). Rates of multiple pregnancy (all were twins) were the same among MWH users and non-users (4.3 %).

**Table 2:** Maternal and fetal risk factors recorded in the Glenn C. Olson Memorial Primary Hospital obstetric logbook, stratified by maternity waiting home (MWH) use, August 2011-March 2014, Yetebon, Ethiopia

Factor	Non-MWH users (*N* = 396), N (%)	MWH users (*N* = 93), N (%)
Maternal age* [median (inter-quartile range)]	27 (24–30)	29 (25–33)
Maternal HIV status (missing = 8)	6 (1.5)	1 (1.1)
Multiple pregnancy	17 (4.3)	4 (4.3)
Previous caesarean section**	21 (5.3)	14 (15.1)
Post-term**	15 (3.8)	20 (21.5)
Fetal malposition/malpresentation	11 (2.8)	5 (5.4)
Placenta previa	1 (0.3)	1 (1.1)
Fetal anomaly (hydrocephalus, anencephaly, ascites)	3 (0.8)	1 (1.1)
Pre-eclampsia	9 (2.3)	3 (3.2)

*P < 0.05, Chi-squared test or Wilcoxon two-sample test

**P < 0.001, Chi-squared test

### Pregnancy outcomes and characteristics of hospital-born infants

MWH users were significantly (*p* < 0.05) more likely than non-users to have a caesarean Sec. [51.1 % (*N* = 47) vs. 35.2 % (*N* = 140)], less likely to have a spontaneous vaginal delivery [48.9 % (*N* = 45) vs. 63.8 % (*N* = 252)], and similarly unlikely to have an assisted delivery [0 % vs. 1 % (*N* = 4)] (Table 3). In addition, MWH users were significantly less likely than non-MWH users to experience obstructed labor [6 of 93 (6.5 %) vs. 57 of 396 (14.4 %)] (*p* < 0.05) or stillbirth [1 of 93 (1.1 %) vs. 34 of 396 (8.6 %)] (*p* < 0.05); there were no cases of uterine rupture among MWH users whereas this occurred in nine women (2.3 % of 396) who were non-MWH users, although the difference was not statistically significant.

**Table 3:** Pregnancy outcomes noted in the Glenn C. Olson Memorial Primary Hospital obstetric logbook, stratified by maternity waiting home (MWH) use, August 2011-March 2014 (*N* = 489), Yetebon, Ethiopia

Outcome	Non-MWH users (*N* = 396), N (%)	MWH users (*N* = 93), N (%)
Caesarean section* (missing = 2)	139 (35.2)	47 (51.1)
Spontaneous vaginal delivery*	252 (63.8)	45 (48.9)
Premature rupture of membranes	4 (1.0)	1 (1.1)
Obstructed or prolonged labor*	57 (14.4)	6 (6.5)
Uterine rupture	9 (2.3)	0 (0.0)
Antepartum hemorrhage	10 (2.5)	1 (1.1)
Postpartum hemorrhage	1 (0.3)	0 (0.0)
Fetal distress	13 (3.3)	1 (1.1)
Intrauterine fetal death	10 (2.5)	2 (2.2)
Stillbirth**	34 (8.6)	1 (1.1)
Live births	413	97
Birthweight (kg)** [median (IQR)] (missing = 8)	3.2 (2.8–3.5)	3.5 (3.0-3.8)
Preterm	7 (1.8)	1 (1.1)
Died after delivery	6 (1.5)	0 (0.0)

*P < 0.05

**P < 0.001, Fisher’s exact text

Among newborns (*N* = 97 including twins) of MWH users, 57.7 % were male and 34.0 % were female, and 8.3 % were not recorded. Among newborns (*N* = 413 including twins) of non-MWH users, 54.5 % were male, 42.9 % were female, and 2.7 % were not recorded. Overall, for children with a recorded sex, the sex ratio was 1.31, with no significant difference in newborn sex distribution between MWH users and non-users (*p* = 0.230).

Among singleton births (*N* = 468) and among all births (*N* = 510; 21 twin pregnancies) to the 489 women who delivered at the hospital, the median birth weight was higher among MWH users (3.5 kg, interquartile range 3.0-3.8) than non-users (3.2 kg, 2.8–3.5) (*p* < 0.001) (Table 3). Eight of the 510 infants born in the hospital were missing birth weight – all were singletons and six were stillborn/intrauterine fetal deaths.

**Fig. 2:**
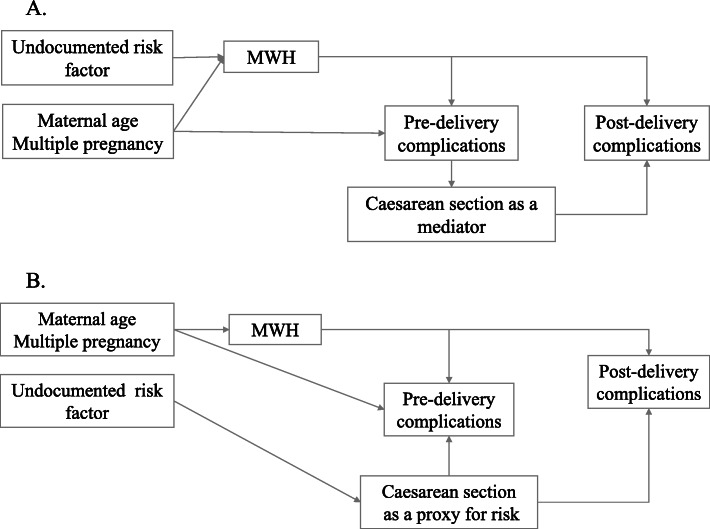
Conceptual framework for relationships between background risk, maternity waiting home use, mode of delivery, and complications. MWH = maternity waiting home. **A**. Caesarean section as a proxy for high-risk pregnancy. **B**. Caesarean section as an outcome and potential mediator

Of 489 women, 126 experienced a childbirth complication; 63 had a complication affecting the fetus or newborn only, 63 had a maternal complication only, and 25 had both a fetal/newborn and a maternal complication). MWH use was associated with a 70 % lower risk of any childbirth complication [unadjusted RR = 0.30, 95 % confidence interval (CI) 0.15–0.58, *p* < 0.001] (model A, Table 4). RR for fetal and newborn complications was 0.06 (95 % CI 0.01–0.7) (*p* = 0.007) and for any maternal complication was 0.37 (95 % CI 0.18–0.77) (*p* < 0.008). Including caesarean section in the models (as per adjusted, conceptual model B, Fig. 2) accentuated the association of MWH with the outcomes. Thus, MWH use was associated with an adjusted 77 % (adjusted RR = 0.23, 95 % CI 0.12–0.46, *p* < 0.001) lower risk of childbirth complications, a 94 % (adjusted RR 0.06, 95 % CI 0.01–0.43, *p* = 0.005) lower risk of fetal and newborn complications, and a 73 % (adjusted RR 0.27, 95 % CI 0.13–0.56, *p* < 0.001) lower risk of maternal complications compared to MWH non-users (model B, Table 4). Notably, caesarean section was associated with a significantly higher risk of any childbirth complication (RR 5.52, 95 % CI 3.85–7.94, *p* < 0.001), fetal and newborn complications (RR 2.52, 95 % CI 1.39–3.99, *p* < 0.001), and any maternal complication (RR 11.29, 95 % CI 6.24–19.72, *p* < 0.001). Increasing maternal age was also associated with increased risk for any fetal complication (RR 1.05, 95 % CI 1.02–1.09, *p* < 0.007). Sensitivity analysis was performed, including complications which were recorded in both the MWH record and obstetric record, and similar results were found (Table S1).

**Table 4:** Factors associated with risk of childbirth complications recorded in the Glenn C. Olson Memorial Primary Hospital obstetric logbook, August 2011-March 2014 (*N*=489), Yetebon, Ethiopia

Factor	Conceptual model A (see Fig. 2)			Conceptual model B (see Fig. 2)		
	Relative Risk	95% Confidence Interval	***p***-value	Relative Risk	95% Confidence Interval	***p***-value
Any complication^a^						
Attended MWH	0.30	0.15 – 0.58	<0.001	0.23	0.12 – 0.46	<0.001
Caesarean section				5.52	3.85 – 7.94	<0.001
Any fetal or newborn complication^a^						
Attended MWH^b^	0.06	0.01– 0.47	0.007	0.06	0.01 – 0.43	0.005
Maternal age (1-year increment)	1.05	1.02 – 1.09	0.007	1.05	1.01 – 1.09	0.005
Caesarean section				2.52	1.39 – 3.99	<0.001
Any maternal complication^c^						
Attended MWH	0.37	0.18 – 0.77	0.008	0.27	0.13 – 0.56	<0.001
Maternal age (1-year increment)				1.03	1.01 – 1.06	0.017
Caesarean section				11.29	6.24 – 19.72	<0.001

^a^Total participants=489, model A missing=8, model A *N*=481; model B missing=9, final model *N*=480

^b^Unadjusted Relative Risk (regression model excludes maternal age)=0.07 (95% CI=0.01 – 0.50)

^c^Total participants=489, model A missing=0; model B missing=2, final model *N*=487

The 1-min Apgar scores were not significantly higher among live births to MWH users (Table 5). The average 5-min Apgar scores were statistically significantly higher among live births to MWH users than non-users (adjusted difference = 0.25, 95 % CI = 0.06–0.44). Caesarean section was associated with significantly lower 1-min (*p* = 0.002) and 5-min (*p* = 0.002) Apgar scores, and multiple pregnancy was associated with lower 1-min Apgar scores (*p* = 0.039).

**Table 5:** Association between factors and Apgar score among live births recorded in the Glenn C. Olson Memorial Primary Hospital obstetric logbook, August 2011-March 2014 (*N* = 489), Yetebon, Ethiopia

Factor	Adjusted Estimate	95 % Confidence interval	*p*-value
1-min Apgar score^a^			
Attended MWH	0.26	-0.02–0.55	0.067
Caesarean section	-0.37	-0.60 – -0.14	0.002
Multiple pregnancy	-0.45	-0.88 – -0.02	0.039
5-min Apgar score^b^			
Attended MWH	0.25	0.06–0.44	0.009
Caesarean section	-0.25	-0.41 – -0.10	0.002

^a^Five hundred ten births in total, 13 missing 1-min Apgar score and an additional 47 with 1-min Apgar score but were intrauterine fetal death/stillbirth. Final model *N* = 450

^b^Five hundred ten births in total, 59 missing 5-min Apgar score and an additional 4 with 5-min Apgar score but were intrauterine fetal death/stillbirth. Final model *N* = 447

### Reasons for use of the MWH: qualitative research

MWH healthcare workers noted that socioeconomic status was the primary determinant of a mother’s interest in a MWH stay:

*Sometimes [despite] the indication to go to the maternity waiting home, the mothers prefer to stay at home. These are mostly coming from the nearby cities. And well-educated. And usually they defer not to get into the maternity waiting home after they visit it, and they found that it is not just like their home. Less comfortable when compared to their home, so, they prefer to stay at home. But for the communities, mothers who are coming from the poor communities, from far rural areas, it is the best place. They want to stay there.* (MWH healthcare worker)

They noted that wealthier women generally used other means to stay “close” to the hospital or to ensure rapid emergency transport to the hospital if needed:

*You know these modern people [laughs] they don’t like to stay here. [If] you have some problems or if you are in labor, just they tell us. They will call us with the telephone, so I give my number and the ambulance number.* (MWH healthcare worker)

All reinforced the necessity of the MWH to mothers’ survival, and the need to upgrade the facility to make it “attractive” to as many women as possible. Healthcare workers noted the importance of governmental Health Extension Workers in promoting maternal health and MWH use, and expressed a desire to collaborate fully across public-private lines with those workers.

Despite touting the merits of MWHs, healthcare workers also acknowledged that other problems existed with MWHs:

*From the big picture side, what’s important is developing the country. That is the only solution. Improving the social status of the community. The infrastructure, education, the way we feed mothers, we feed children… when compared to this maternity waiting home—the maternity waiting home is nothing at all. The problem in Ethiopia is poverty. (MWH healthcare worker)*

MWH users said that they would stay there again if necessary and had few complaints about the facility. They noted that, when accompanied by their husbands, both were unable to work, which was a financial hardship. Additionally, someone else – frequently a mother-in-law – had to care for children left at home. Lack of water at the MWH was noted to be the major challenge to living there – water had to be carried from the hospital. They also noted that while the stay at the MWH and care in the hospital were free-of-charge, they had to purchase some meals. Both MWH users said that it would take 5 h to several days to return home from the MWH, which they would do on foot after delivery, as soon as they were physically able.

Another aspect of the MWH that both providers and users mentioned was the lack of available activity. Providers noted that it would be nice for women to have a television or some form of entertainment, and both users could not name anything in particular that they were doing while at the MWH.

## Discussion

Women used the Project Mercy MWH most commonly due to post-term pregnancy status, prior history of caesarean section or myomectomy, and complications associated with the placenta or with the position of the baby in utero. Overall, about three-fourths of women who stayed in the MWH gave birth in the hospital, and MWH users were three-fold more likely than non-users to have had a caesarean section and five-fold more likely to be post-term. MWH users were more likely than non-users to undergo caesarean section or experience obstructed labor or stillbirth. Overall, the risks for any childbirth complication, for fetal and newborn complications, and for maternal complications were significantly lower – by 78 %, 94 %, and 73 %, respectively – among MWH users compared to non-users. In addition, birth weight and 5-min Apgar scores were significantly higher among MWH users compared to non-users.

Healthcare providers and MWH users interviewed for this study identified socioeconomic implications as a primary deterrent for MWH usage. There was a suggestion that use of the MWH was associated with being impoverished, raising the potentially that it was stigmatizing, although this requires further research. Women users of the MWH underscored the importance of family support in making the journey with them and helping watch children left at home, frequently mentioning the challenge of long travel times. They also communicated the need for increased availability of food, water, and entertainment in MWHs. While universally agreeing that MWH quality needed to improve to attract more users, women who used the MWH generally reflected their willingness to return for subsequent pregnancies.

Many quantitative and qualitative themes emerged in this locally specific study which paralleled those in other contexts throughout the world. Others have similarly found correlation between MWH use and improvement in various measures of birth outcomes including decreases in obstructed labor [5, 30, 31], stillbirth [5, 9, 26, 30], and uterine rupture [5, 13, 25, 30, 32], as well as higher neonatal assessment scores (e.g. Apgar) and perinatal and maternal mortality [26, 30]. These findings are significant, because of the potential benefits of MWH use for women at high risk of obstetric complications. However, these results also need to be interpreted with caution because of the heterogeneity of study contexts, and in many studies confounding factors were not taken into account. Our study sample had an increased rate of caesarean section among MWH users, which was consistent with the findings of several similar studies of MWHs [9, 13, 31, 32]. This may result from pregnancy complications or prior history which necessitated the access to facility care afforded by MWHs. Moreover, caesarean section across all pregnancies was associated with upwards of a five-fold, significantly increased risk for any complication or a fetal complication. In low- and middle-income countries, caesarean section is associated with higher morbidity and mortality rates and an increased risk of childbirth complications when compared with vaginal delivery [33–36]. Our data showing improved outcomes among MWH users despite their higher proportion of caesarean section (model A results) may reflect the preparatory nature of MWH attendance in the context of low national rates of skilled birthing attendance, with direct hospital admission occurring more often in the event of unplanned complications and emergency obstetric care.

Many qualitative themes, including financial concerns and lack of MWH infrastructure (such as water access), were also present in existing research [37]. A 2019 study found that MWH users paid twice as much out-of-pocket as women admitted directly to healthcare facilities [8]. Our interviews confirmed that, although governmental measures have decreased the cost of facility delivery, additional expenses associated with MWH use may prevent families from utilizing them. The difficult and long journey to MWHs was another frequently mentioned obstacle to their use. A 2017 World Bank estimate suggests that 79 % of Ethiopians live in rural areas, increasing both the potential benefits and challenges associated with MWH use [38]. Rural Ethiopian women experience the highest rates of obstetric complications [5], and further research is required to determine whether providing viable transportation options to these women would increase MWH utilization and access to skilled birth attendance and quality emergency obstetric care in health facilities.

This study shares many of the challenges present in other MWH research, including its retrospective nature, limited sample size, and lack of randomization and MWH standardization. It lacked contextual information such as the analogous complication rates among women not utilizing facilities or MWHs [27]. We were unable to systematically assess potential bias in our study sample, as the MWH users and non-users were self-selected. While we adjusted for important measured confounders, there could still be other confounding factors that were unmeasured. The results from the low-volume rural community hospital where we conducted the study (489 births over 32 months) should be viewed as indicative and may not be generalizable to higher-volume settings. The MWH logbook did not record socio-cultural, economic or logistical factors in MWH use, and the limited number of qualitative interviews – and none among non-users of the MWH – were selected by convenience and did not enable information saturation to be achieved. Thus, insights into non-biomedical reasons for MWH use were directional yet limited.

 This study’s strengths include the detailed nature of the data, and the characterization of a single MWH in a local context both qualitatively and quantitatively, providing highly relevant reflections to the team of healthcare providers and the community they serve. In addition to the consideration of health impacts, local context – which is by definition specific and non-standardized – may be an equally important contributor to understanding the function and benefits of MWHs. For example, the providers we interviewed used this awareness to predict which patients would benefit most from MWHs beyond medical or geographic indication (for example, which women could stay in touch by cellphone). A 2019 study also noted that healthcare workers can play a critical role in community health education because of their unique ability to effectively personalize their approach and delivery [39]. This local knowledge might never factor into standardized guidelines regarding MWH referral, but is nonetheless important to consider.

## Conclusions

Our study highlights significant potential health benefits from use of a MWH, as well as important shortcomings in data on MWH effectiveness and use. We note several additional areas in which further research is needed, including greater understanding of barriers and facilitating factors in MWH utilization. Opportunity and out-of-pocket costs of MWH use, potential stigma associated with MWH use, and transportation options and engagement of frontline workers to facilitate MWH utilization require further investigation. Beyond a focus on the medical benefits of MWHs, it may be valuable to consider their worth as more than simply a place to “wait.” As other research has suggested, if communities have a stake in designing MWHs, they may serve as centers for women’s empowerment, education, and income generation, impacting women and families far beyond birth outcomes [24, 28]. If MWHs could be leveraged in this fashion, the ongoing debate over their medical efficacy may become a smaller component of their assessed “worth” as an intervention [40].

## Supplementary Information

**Additional file 1: Table S1.** Complications relative risk based on Glenn C. Olson Memorial Primary Hospital obstetric logbook regardless of a prior record of the same complication in the maternity waiting home (MWH) logbook.

## Data Availability

The dataset generated and analyzed during the current study is not publicly available as it was derived from patient medical records, but the data are available from the corresponding author on reasonable request.

## Abbreviations

CI: Confidence interval
HIV: Human immunodeficiency virus
MWH: Maternity waiting home
RR: Relative risk

## References

[1] Kyei-Nimakoh M, Carolan-Olah M, McCann TV (2017). Access barriers to obstetric care at health facilities in sub-Saharan Africa—a systematic review. Syst Rev..

[2] Maternal mortality ratio (Modeled estimate, per 100,000 live births) - Ethiopia | Data. Available from: https://data.worldbank.org/indicator/SH.STA.MMRT?locations=ET. [cited 28 Aug 2020]

[3] Thaddeus S, Maine D (1994). Too far to walk: maternal mortality in context. Soc Sci Med.

[4] Tesfaye B, Mathewos T, Kebede M (2017). Skilled delivery inequality in Ethiopia: to what extent are the poorest and uneducated mothers benefiting?. Intl J Equity Health..

[5] Meshesha B, Dejene G, Tesfahun H. The role of maternity waiting area in improving obstetric outcomes: a comparative cross-sectional study, Jinka Zonal Hospital, Southern regional state. J Women’s Health Care. 2017;06(06). Available from: https://www.omicsonline.org/open-access/the-role-of-maternity-waiting-area-in-improving-obstetric-outcomes-a-comparative-crosssectional-study-jinka-zonal-hospital-souther-2167-0420-1000406-96933.html [cited 2020 Aug 28]

[6] Ministry of Health Ethiopia. Guideline for the establishment of standardized maternity waiting homes at health centres/facilities. Addis Ababa: Federal Ministry of Health (FMOH); 2015.

[7] Ethiopian Public Health Institute, Averting Maternal Death and Disability. Ethiopian emergency obstetric and newborn care (EmONC) assessment 2016: Final report. Addis Ababa: Ethiopian Public Health Institute; 2017.

[8] Getachew B, Liabsuetrakul T. Health care expenditure for delivery care between maternity waiting home users and nonusers in Ethiopia. Intl J Health Plann Manage. 2019;34(2):e1334–45. 10.1002/hpm.2782 30924204

[9] Getachew B, Liabsuetrakul T, Gebrehiwot Y (2020). Association of maternity waiting home utilization with women’s perceived geographic barriers and delivery complications in Ethiopia. Intl J Health Plan Manage.

[10] Hibstu DT, Siyoum YD. Knowledge of obstetric danger signs and associated factors among pregnant women attending antenatal care at health facilities of Yirgacheffe town, Gedeo zone, Southern Ethiopia. Arch Public Health. 2017;75. Available from: https://www.ncbi.nlm.nih.gov/pmc/articles/PMC5554969/ [cited 28 Aug 2020]. 10.1186/s13690-017-0203-y PMC5554969 28811893

[11] Wong KLM, Benova L, Campbell OMR (2017). A look back on how far to walk: Systematic review and meta-analysis of physical access to skilled care for childbirth in Sub-Saharan Africa. PLoS ONE.

[12] Henry EG, Semrau K, Hamer DH, Vian T, Nambao M, Mataka K, et al. The influence of quality maternity waiting homes on utilization of facilities for delivery in rural Zambia. Reprod Health. 2017;14. Available from: https://www.ncbi.nlm.nih.gov/pmc/articles/PMC5450262/. [cited 28 Aug 2020] 10.1186/s12978-017-0328-z PMC5450262 28558800

[13] Braat F, Vermeiden T, Getnet G, Schiffer R, van den Akker T, Stekelenburg J. Comparison of pregnancy outcomes between maternity waiting home users and non-users at hospitals with and without a maternity waiting home: retrospective cohort study. Intl Health. 2018 01;10(1):47–53. 10.1093/inthealth/ihx056 29342256

[14] Vermeiden T, Braat F, Medhin G, Gaym A, van den Akker T, Stekelenburg J (2018). Factors associated with intended use of a maternity waiting home in Southern Ethiopia: a community-based cross-sectional study. BMC Pregnancy Childbirth.

[15] Kebede KM, Mihrete KM. Factors influencing women’s access to the maternity waiting home in rural Southwest Ethiopia: a qualitative exploration. BMC Pregnancy Childbirth. 2020;20. Available from: https://www.ncbi.nlm.nih.gov/pmc/articles/PMC7226938/ [cited 2020 Aug 28]. 10.1186/s12884-020-02988-8 PMC7226938 32408875

[16] Singh K, Speizer I, Kim ET, Lemani C, Phoya A (2017). Reaching vulnerable women through maternity waiting homes in Malawi. Intl J Gynecol Obstet.

[17] Windsma M, Vermeiden T, Braat F, Tsegaye AM, Gaym A, van den Akker T, et al. Emergency obstetric care provision in Southern Ethiopia: a facility-based survey. BMJ Open. 2017;7(11). Available from: https://www.ncbi.nlm.nih.gov/pmc/articles/PMC5695514/ [cited 2020 Aug 28] 10.1136/bmjopen-2017-018459 PMC5695514 29122802

[18] Mgawadere F, Unkels R, Kazembe A, van den Broek N (2017). Factors associated with maternal mortality in Malawi: application of the three delays model. BMC Pregnancy Childbirth..

[19] Geleto A, Chojenta C, Musa A, Loxton D (2018). Barriers to access and utilization of emergency obstetric care at health facilities in sub-Saharan Africa: a systematic review of literature. Syst Rev..

[20] Vermeiden T, Stekelenburg J (2017). Maternity waiting homes as part of an integrated program for maternal and neonatal health improvements: women’s lives are worth saving. J Midwifery Womens Health.

[21] Jackson R, Tesfay FH, Gebrehiwot TG, Godefay H (2017). Factors that hinder or enable maternal health strategies to reduce delays in rural and pastoralist areas in Ethiopia. Trop Med Intl Health.

[22] Sialubanje C, Massar K, Kirch EM, Pijl MSG van der, Hamer DH, Ruiter RAC. Husbands’ experiences and perceptions regarding the use of maternity waiting homes in rural Zambia. Intl J Gynecol Obstet. 2016;133(1):108–11. 10.1016/j.ijgo.2015.08.023 26873126

[23] Chibuye PS, Bazant ES, Wallon M, Rao N, Fruhauf T (2018). Experiences with and expectations of maternity waiting homes in Luapula Province, Zambia: a mixed–methods, cross-sectional study with women, community groups and stakeholders. BMC Pregnancy Childbirth..

[24] Bergen N, Abebe L, Asfaw S, Kiros G, Kulkarni MA, Mamo A (2019). Maternity waiting areas – serving all women? Barriers and enablers of an equity-oriented maternal health intervention in Jimma Zone, Ethiopia. Global Public Health.

[25] Vermeiden T, Schiffer R, Langhorst J, Klappe N, Asera W, Getnet G (2018). Facilitators for maternity waiting home utilisation at Attat Hospital: a mixed-methods study based on 45 years of experience. Trop Med Intl Health.

[26] Dadi TL, Bekele BB, Kasaye HK, Nigussie T (2018). Role of maternity waiting homes in the reduction of maternal death and stillbirth in developing countries and its contribution for maternal death reduction in Ethiopia: a systematic review and meta-analysis. BMC Health Services Res..

[27] van Lonkhuijzen L, Stekelenburg J, van Roosmalen J (2012). Maternity waiting facilities for improving maternal and neonatal outcome in low-resource countries. Cochrane Database Systematic Rev.

[28] Lori JR, Munro-Kramer ML, Mdluli EA, Musonda Mrs GK, Boyd CJ (2016). Developing a community driven sustainable model of maternity waiting homes for rural Zambia. Midwifery..

[29] Glaser BG, Strauss AL (2009). The discovery of grounded theory: strategies for qualitative research. 4. paperback printing.

[30] Tiruneh GT, Getu YN, Abdukie MA, Eba GG, Keyes E, Bailey PE (2019). Distribution of maternity waiting homes and their correlation with perinatal mortality and direct obstetric complication rates in Ethiopia. BMC Pregnancy Childbirth..

[31] Chandramohan D, Cutts F, Chandra R (1994). Effects of a maternity waiting home on adverse maternal outcomes and the validity of antenatal risk screening. Intl J Gynaecol Obstet.

[32] Kelly J, Kohls E, Poovan P, Schiffer R, Redito A, Winter H (2010). The role of a maternity waiting area (Mwa) in reducing maternal mortality and stillbirths in high-risk women in rural Ethiopia. BJOG..

[33] Sobhy, S, Arroyo-Manzano, D, Murugesu, N, Karthikeyan, G, Kumar, V, Kaur, I, et al. Maternal and perinatal mortality and complications associated with caesarean section in low-income and middle-income countries: a systematic review and meta-analysis. Lancet. 2019;10184:1973–82. 10.1016/S0140-6736(18)32386-9 30929893

[34] Souza JP, Gulmezoglu AM, Vogel J, Carroli G, Lumbiganon P, Qureshi Z, et al. Moving beyond essential interventions for reduction of maternal mortality (the WHO Multicountry Survey on Maternal and Newborn Health): A cross-sectional study. Lancet. 2013;9879:1747–55. 10.1016/S0140-6736(13)60686-8 23683641

[35] Epiu I, Tindimwebwa JVB, Mijumbi C, Chokwe TM, Lugazia E, Ndarugirire F, et al. Challenges of anesthesia in low- and middle-income countries: a cross-sectional survey of access to safe obstetric anesthesia in East Africa. Anesth Analg. 2017;124:290–9. 10.1213/ANE.0000000000001690 PMC5767165 27918334

[36] Sandall J, Tribe RM, Avery L, Mola G, Visser GHA, Homer CSE, et al. Short-term and long-term effects of caesarean section on the health of women and children. Lancet. 2018;10155:1349–57. 10.1016/S0140-6736(18)31930-5 30322585

[37] Tiruneh GT, Taye BW, Karim AM, Betemariam WA, Zemichael NF, Wereta TG (2016). Maternity waiting homes in rural health centers of Ethiopia: the situation, women’s experiences and challenges. Ethiopian J Health Dev.

[38] Rural population (% of total population) - Ethiopia | data. Available from: https://data.worldbank.org/indicator/SP.RUR.TOTL.ZS?locations=ET [cited 28 Aug 2020]

[39] Asfaw S, Morankar S, Abera M, Mamo A, Abebe L, Bergen N (2019). Talking health: trusted health messengers and effective ways of delivering health messages for rural mothers in Southwest Ethiopia. Arch Public Health.

[40] Grown C, Gupta GR, Pande R (2005). Taking action to improve women’s health through gender equality and women’s empowerment. Lancet.

