# Supplementary material for: Association between maternity waiting home stay and obstetric outcomes in Yetebon, Ethiopia: a mixed-methods observational cohort study
Source: BMC Pregnancy Childbirth. 2021 Jul 3;21:482. doi: 10.1186/s12884-021-03913-3 (PMC8254337; doi:10.1186/s12884-021-03913-3)
Supplement: Supplementary file 1 — Additional file 1: Table S1. Complications relative risk based on Glenn C. Olson Memorial Primary Hospital obstetric logbook regardless of a prior record of the same complication in the maternity waiting home (MWH) logbook. [file 12884_2021_3913_MOESM1_ESM.docx]

**Association between maternity waiting home stay and obstetric outcomes in Yetebon, Ethiopia: a mixed-methods observational cohort study**

Anne K Erickson, Safa Abdalla, Alice Serenska, Bete Demeke, and Gary L Darmstadt

**Supplementary material**

**Table S1. Complications relative risk based on Glenn C. Olson Memorial Primary Hospital obstetric logbook regardless of a prior record of the same complication in the maternity waiting home (MWH) logbook**

| **Factor** | **Conceptual model A (see Figure 2)** | | | | | | **Conceptual model B (see Figure 2)** | | | | | | | | |  |  |
| --- | --- | --- | --- | --- | --- | --- | --- | --- | --- | --- | --- | --- | --- | --- | --- | --- | --- |
|  | **Relative Risk** | **95% Confidence Interval** | | **p-value** | | | **Relative Risk** | | **95% Confidence Interval** | | | **p-value** | | | |  |  |
|  | *Any complication** | | | | | | | | | | | | | | | | |
| Attended MWH | 0.44 | | 0.26 – 0.77 | | | 0.004 | | 0.35 | | | 0.20 – 0.61 | | | <0.001 | | |  |
| Caesarean section |  | |  | |  | | 5.20 | | | 3.65 – 7.51 | | | <0.001 | | |  |  |
| *Any fetal or newborn complication** | | | | | | | | | | | | | | | |  |  |
| Attended MWH** | 0.27 | | 0.10 – 0.73 | | 0.010 | | 0.24 | | | 0.09 – 0.67 | | | 0.006 | | |  |  |
| Maternal age (1-year increment) | 1.04 | | 1.00 – 1.08 | | 0.030 | | 1.04 | | | 1.02 – 1.08 | | | 0.020 | | |  |  |
| Caesarean section |  | |  | |  | | 2.35 | | | 1.49 –3.69 | | | <0.001 | | |  |  |
| *Any maternal complication**** | | | | | | | | | | | | | | |  |  |  |
| Attended MWH | 0.42 | | 0.21 – 0.84 | | 0.014 | | 0.31 | | | 0.16 – 0.61 | | | <0.001 | | |  |  |
| Maternal age (1-year increment) |  | |  | |  | | 1.03 | | | 1.01 – 1.06 | | | 0.019 | | |  |  |
| Caesarean section |  | |  | |  | | 11.18 | | | 6.29 – 19.87 | | | <0.001 | | |  |  |

*Total participants = 489, model A missing = 8, model A N = 481; model B missing = 9, final model N = 480

**Unadjusted RR (regression model excludes maternal age) = 0.28 (0.10 – 0.75)

***Total participants = 489, model A missing = 0; model B missing = 2, final model N = 487
